# Influence of Rice Physicochemical Properties on High-Quality Fresh Wet Rice Noodles: Amylose and Gel Consistency as Key Factors

**DOI:** 10.3390/gels11090696

**Published:** 2025-09-02

**Authors:** Dezhi Zhao, Yuanyuan Deng, Qi Huang, Guang Liu, Yan Zhang, Xiaojun Tang, Pengfei Zhou, Zhihao Zhao, Jiarui Zeng, Ying Liu, Ping Li

**Affiliations:** 1 College of Biotechnology, Tianjin University of Science & Technology, Tianjin 300457, China; zdzyyds6666@163.com; 2 Sericultural & Agri-Food Research Institute Guangdong Academy of Agricultural Sciences, Key Laboratory of Functional Foods, Ministry of Agriculture and Rural Affairs, Guangdong Key Laboratory of Agricultural Products Processing, Guangzhou 510610, China; yuanyuan_deng@sohu.com (Y.D.); liuguang@gdaas.cn (G.L.); zhangyanhb@tom.com (Y.Z.); xjtang66@163.com (X.T.); zhoupengfei@gdaas.cn (P.Z.); zhaozhihao1991@163.com (Z.Z.); zengjiarui@hotmail.com (J.Z.); 3 College of Food Science and Technology, Huazhong Agricultural University, Wuhan 430070, China; 15367983808@163.com

**Keywords:** rice, fresh wet rice noodles, amylose content, gel consistency, sensory evaluation, texture characteristics

## Abstract

Fresh wet rice noodles (FWRNs) are a popular staple food in southern China. The quality of rice varieties results in the inconsistent quality of FWRNs. However, evaluation of rice adaptability for the production of FWRNs is not comprehensive due to the absence of unified screening standards. In this study, twelve rice varieties in southern China were selected to analyze the correlations between rice’s physicochemical properties and the quality characteristics of FWRNs. Results showed that KIM, GC, and IZG rice exhibited a high chalky grain rate and low gel consistency, while the related starches had a high amylose content, high setback value, and low short-range order. Their noodles achieved high total sensory scores and exhibited high levels of sensory and textural qualities. Correlation analysis revealed that the chalky grain rate, chalkiness degree, protein and fat contents, and amylose content were significantly and positively correlated with the hardness, elasticity, chewiness, and total sensory score of FWRNs. Therefore, based on the structural parameters of KIM and GC rice, amylose content between 26–28% and gel consistency between 33–36 mm would be the key factors for raw rice to make high-quality FWRNs. These results offer theoretical guidance for rice selection in the industrial-scale production of FWRNs.

## 1. Introduction

Rice is one of the most important staple foods globally, with China being both a major producer (accounting for approximately 28% of global production [1]) and consumer. As an important rice processing food, rice noodles have become an important part of the catering industry in southern China due to their convenience, reasonable nutritional value, and rich taste. Rice noodle production involves the processes of soaking and grinding the rice, gelatinization of the starch component by heating, and/or extrusion of the resulting product into the desired noodle shape [2]. Depending on the processing methods and formulations, rice noodles can be classified as fresh wet, dried, or fried instant noodles [3].

Fresh wet rice noodles (FWRNs), with steamed vermicelli roll and kway teow as representatives in southern China, are made through a series of processes, including cleaning, soaking, grinding, gelatinization, and cutting into strips. These kinds of FWRNs are produced from rice as the primary ingredient and have a moisture content ranging from 32% to 40%. Compared with dried rice noodles, fresh wet rice noodles (FWRNs) have a smooth texture and are easier to chew due to their high moisture content. Rice quality directly determines the taste and quality of rice noodles [4]. Previous studies have reported that the quality of rice noodles is largely affected by the amylose content, gelatinization temperature, and protein composition of the rice variety [5]. It is widely accepted that rice noodles with a higher amylose content tend to have an increased hardness, while the gelatinization temperature influences the structure of the starch gel network. There is a significant positive correlation between the degree of gelatinization and the breakage and cooking loss rates of rice noodles [6,7]. Furthermore, the protein content is often positively correlated with the taste, hardness, and chewiness of FWRNs, as the interaction between protein and starch results in a more compact and stable gel structure in the rice noodles [8]. However, previous studies have failed to systematically and comprehensively evaluate the adaptability of rice raw materials for the production of FWRNs, particularly overlooking the synergistic effects of the variety of physicochemical characteristics of rice, resulting in the absence of unified screening standards.

In recent years, more research has begun to focus on the characteristic screening of rice to produce high-quality rice noodles. For instance, Cham et al. found that rice with an amylose content of 24% to 27% was suitable for the production of FWRNs [9]. Furthermore, the chalkiness of rice serves as an indicator of its appearance quality and is highly correlated with amylose content in the prediction of the quality of rice noodles [10]. However, there are many varieties of rice in China, which are vulnerable to regional and climatic influences. The quality of these rice varieties differs considerably, resulting in the inconsistent quality of FWRNs [5,11,12]. Therefore, it is fundamentally important to elucidate the effects of the characteristics of different rice varieties on the quality of FWRNs. The objectives of the present study were to (1) analyze the basic composition and physicochemical properties of 12 representative rice varieties produced in southern China, as well as the structural characteristics of the related rice starches; (2) comprehensively evaluate the sensory, textural, and cooking qualities of FWRNs produced from each rice variety; and (3) discuss the correction of rice quality (including composition and physicochemical properties) and FWRN quality (sensory and textural). The findings of this study provide theoretical and practical references for raw rice material selection for FWRNs.

## 2. Results and Discussion

### 2.1. The Composition and Gel Consistency of Different Rice Varieties

#### 2.1.1. The Composition of Different Rice Varieties

The moisture content, chalky grain rate, chalkiness, protein content, fat content, amylose content, and total starch content of the different rice varieties are shown in Table 1. The total starch content among the different rice varieties ranged from 80.83% to 90.44%, with Kaima (KIM) ranking the highest, while the chalky grain rate ranged from 14.73% to 81.27% and chalkiness levels from 4.13% to 35.33%. The chalkiness rate and chalkiness of India Zhengui (IZG) rice were the lowest at 14.73% and 4.13%, respectively. In contrast, KIM and Guichao (GC) rice had a relatively high chalky grain rate and chalkiness at 82.53% and 35.43% and 81.87% and 34.53%, respectively. There were no significant differences among most of the rice varieties. The amylose content of different rice varieties was between 14.61% and 27.66%, with KIM and GC rice having the highest amylose levels at 27.66% and 26.81%, respectively, while GSLS rice had the lowest at 14.61%. These results confirm that rice chalkiness is highly correlated with amylose content, consistent with a previous report [10]. The protein content of the rice varieties ranged from 5.48% to 8.98%, with YZ and GC rice having the highest protein levels (8.98% and 8.31%, respectively). The fat content in the selected rice samples was relatively low, varying between 0.08% and 0.84%.

#### 2.1.2. Gel Consistency of the Rice Varieties

The gel consistency indicates the viscoelastic properties of rice flour following rehydration, as measured by the extensibility of the rice gel. Rice gel can be categorized into three types based on the flow length: soft (>60 mm), medium (40 to 60 mm), and hard (<40 mm). The gel consistency results of the different rice varieties are shown in Table 2, ranging from 33.97 to 77.33 mm. KIM, GC, JC, and JCX rice had relatively low gel consistency (33.97–37.77 mm), which could be attributed to the higher amylose content [13]. Conversely, GSLS, JG, WEA, IZG, and YZ rice had high gel consistency (60.37–77.33 mm). Among all varieties, GSLS rice had the highest gel consistency (77.33 mm). The inverse relationship between amylose content and gel consistency could be explained by the fact that lower amylose content typically results in less rigid molecular structures and greater water retention capacity. Amylose is the main polymer that leaches as starch granules are heated, and it forms networks as the gel starts to cool. As amylose molecules tend to form strong helical structures that limit water absorption, varieties with lower amylose (like GSLS) generally exhibit higher gel consistency due to greater swelling power and more flexible gel networks. This observation aligns with previous findings by Singh [14], who reported similar inverse correlations in rice starches.

### 2.2. Structural Characteristics of Rice Starches from Different Rice Varieties

#### 2.2.1. Pasting Properties

**Figure 1:**
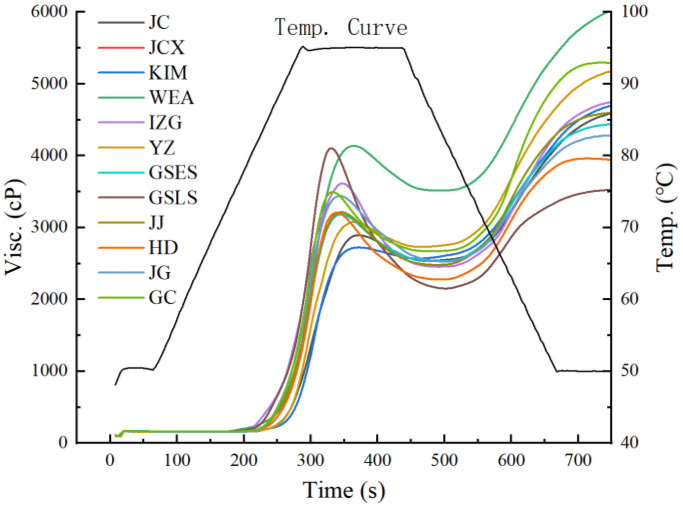
Pasting property profile of starches from different rice varieties. Different rice varieties: Jicheng (JC), Jinchangxiang (JCX), Wang’er’ai (WEA), Yuanzao (YZ), Kaima (KIM), India Zhengui (IZG), Guishan early-season (GSES), Guishan late-season (GSLS), Jiajia (JJ), Hengda (HD), Jingu (JG).

The pasting properties of different rice starches are shown in Figure 1 and Table 2. There were significant differences in the pasting properties of different rice starches. KIM and IZG rice starches were gelatinized at relatively low temperatures (79.52 and 80.52 °C, respectively) and had relatively high peak viscosities (3530.00 and 3603.67 cP, respectively), indicating easier disruption of hydrogen bonds within the starch [15]. However, JC and YZ rice starches had the highest gelatinization temperatures (86.22 and 86.97 °C, respectively) and the lowest peak viscosities (2889.33 and 2726.67 cP, respectively). These results could be attributed to their higher protein content (7.46% and 8.98%), which would restrict expansion of the starch granules when heated. WEA rice starch had the highest peak viscosity (4104.33 cP) and trough viscosity (3473.00 cP), indicating the greatest resistance to water absorption and swelling capacity. Correspondingly, GSLS rice starch had the highest peak viscosity (4115.00 cP) and breakdown value (1922.33 cP), which indicated the poorest structural stability. As illustrated in Figure 1, all rice starches reached the final viscosity during the cooling stage (50 °C). The WEA rice starch had the highest final viscosity (5884.67 cP), potentially due to the formation of a stable gel network structure upon cooling, which enhances viscosity [16]. The setback values of the GC and KIM rice starches were notably the highest due to the greater amylose content. Conversely, GSLS rice starch, with the lowest amylose content, had the smallest setback value (1358.67 cP), suggesting the strongest resistance to retrogradation. This result differed from the highest gel consistency value of GSLS. This could be attributed to the fact that the pasting properties primarily reflect starch behavior during heating and shearing, while gel consistency measures cold gel viscosity. Factors like amylopectin branch chain length distribution and molecular weight may affect these properties differently. For instance, certain long-branched amylopectin structures could contribute to poor thermal stability (affecting pasting) while still forming strong gels upon cooling. This observation aligns with previous findings reported by Li [17].

#### 2.2.2. The Relative Crystallinity and Short-Range Order of Different Rice Starches

**Figure 2:**
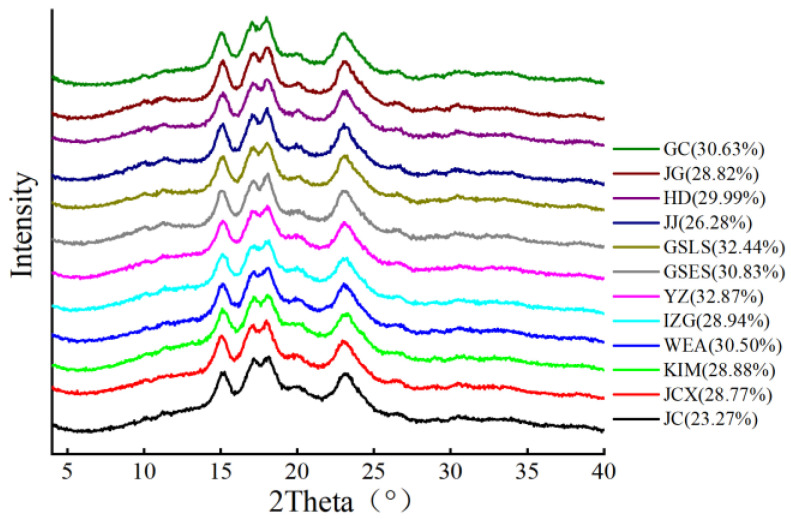
X-ray diffraction profile and relative crystallinity of starch from different rice varieties. Different rice varieties: Jicheng (JC), Jinchangxiang (JCX), Wang’er’ai (WEA), Yuanzao (YZ), Kaima (KIM), India Zhengui (IZG), Guishan early-season (GSES), Guishan late-season (GSLS), Jiajia (JJ), Hengda (HD), Jingu (JG), Guichao (GC).

The relative crystallinity profile and parameters of different rice starches are shown in Figure 2. All rice starches exhibited the A-type polymorph, with the main reflections at 15°, 17°, 18°, and 23° 2θ. Most rice starch samples had weak diffraction peaks near 20° 2θ, which could be attributed to the formation of starch–lipid complexes [18]. As illustrated in Figure 2, the relative crystallinity of all rice starches varied from 23.27% to 32.87%. The YZ and GSLS rice starches had the highest relative crystallinity (32.87% and 32.44%, respectively), whereas that of JC ranked the lowest. This result was partially consistent with the amylose content (Table 2). In general, the relative crystallinity could be attributed to crystal size, the number of crystalline regions (influenced by amylopectin content and the chain length distribution of amylopectin), the orientation of double helices within the crystalline domains, and the extent of interactions among the double helices [19].

The infrared spectra (IR) absorbance band at 1045 cm^−1^ is sensitive to the amount of ordered or crystalline structures in starch, and the band at 1022 cm^−1^ is characteristic of the amorphous structure of starch. Thus, the bands at 1045 and 1022 cm^−1^ have been associated with ordered and amorphous structures in starch, respectively. The absorbance ratio can be used as an index to characterize the short-range alignment of helices, where the larger the ratio is, the higher the degree of short-range order in starch granules [20]. As shown in Table 3, the short-range order of the KIM and GC rice starches was relatively low (2.13 and 2.14, respectively) compared to the starch counterparts, consistent with the relative crystallinity [19]. However, GSLS rice starch exhibited higher ratios at 1045 and 1022 cm^−1^, which may be due to the greater number of crystalline regions of double helices formed by amylopectin [21], consistent with the relatively high crystallinity and low amylose content.

#### 2.2.3. Thermal Properties of Different Rice Starches

Thermal properties can determine the process of ordered structure disruption in starch during gelatinization, thereby reflecting thermodynamic changes. Starch gelatinization is an endothermic transition that corresponds to the dissociation of amylopectin double helices from a semi-crystalline structure to an amorphous conformation. The thermodynamic properties of different rice starches are shown in Table 3. The thermal temperature ranged from 64.51 to 87.57 °C and the enthalpy changes from 8.55 to 14.65 J/g. WEA rice starch had the lowest thermal temperatures (*T*_o_, *T*_p_, and *T*_c_), whereas the JC rice starch had the highest. KIM and GC rice starches had the highest thermal temperature (To) at 73.41 and 72.31 °C, respectively. These results were not totally consistent with the pasting properties of rice (Table 2), which could be attributed to the fact that thermal properties are largely affected by the fine structure of the amylopectin and amylose contents in different rice starches. In general, starches consisting of amylopectin with longer branch chains have higher gelatinization temperatures due to the formation of stable double helical crystallites. The JG rice starch had the highest Δ*H* value, which was nearly 25% higher than those of the KIM, IZG, JJ, and GSES rice starches. This result could possibly be attributed to the highly ordered starch structure and double helical structures. Hence, the thermal process would require more energy to completely destroy the crystal structure [22]. Notably, GSLS and WEA rice starches had higher relative crystallinity but the lowest enthalpy changes. Enthalpy change is a combination of the endothermic disruption process of the ordered structure and the exothermic process of granular swelling [23], with enthalpy changes primarily reflecting loss of the double helical order rather than the crystalline structure [24].

### 2.3. Quality Evaluation of FWRNs Prepared from Different Rice Varieties

#### 2.3.1. Appearance and Cooking Loss Rate of FWRNs Prepared from Different Rice Varieties

The color of FWRNs is the initial visual impression of consumers and largely influences preferences. The L* value of rice noodles, as measured by a spectrophotometer, indicates lightness and darkness, with positive a* values representing redness and negative a* values indicating greenness, while positive b* values signify yellowness and negative b* values denote blueness. The brown index (BI) is also evaluated according to the L*, a*, and b* values. Consumers frequently prefer bright, shiny FWRNs, which are characterized by high lightness (L* value) and low yellowness (b* value) [25] as well as low BI. As indicated in Table 4, there were significant color differences among the FWRNs produced from the 12 rice varieties. FWRNs made from YZ, GC, and IZG rice had higher L* values (75.04, 74.59, and 73.97, respectively) than the WEA and HD rice (66.52 and 67.69, respectively). The b* values of FWRNs made from YZ and JC rice (−0.49 and −0.80, respectively) were higher, while the yellowness value of GSLS rice was the lowest. Rice varieties with lower amylose content (e.g., GSLS) tend to produce noodles with higher L* (lightness) due to reduced retrogradation and a more uniform gel structure, minimizing light scattering [26]. In addition, Maillard reactions (non-enzymatic browning) between reducing sugars and proteins can increase yellowness (b*) [27]. Varieties with higher protein content (e.g., JC) may develop more yellowish hues during processing. Lipids in rice could also oxidize during milling and noodle preparation, contributing to yellow color changes. It is worth noting that FWRNs made from JC and YZ rice possessed the highest BI values among all the samples. The KIM, IZG, GSLS, and GC rice noodles exhibited negative BI values, indicating the lowest degree of browning. Therefore, FWRNs made from IZG, GC, and GSLS rice present a brighter white appearance.

As shown in Table 4, the cooking loss rates of FWRNs made from different rice varieties were all relatively low (0.07–0.49%). Among all samples, FWRNs prepared from GSLS rice had the highest cooking loss rate (0.49%), which could be attributed to this rice having the lowest amylose content and because the gel structure formed by rice starch with low amylose content is easily dissolved due to weak restriction forces.

#### 2.3.2. Texture of FWRNs Prepared from Different Rice Varieties

The taste and tissue structure of FWRNs are important indicators of texture. Consequently, hardness, adhesiveness, elasticity, and chewiness serve as texture indices that represent the quality of FWRNs to a certain extent. The texture profiles of FWRNs varied significantly across rice varieties (Table 5), reflecting differences in starch and protein composition as well as processing-induced microstructural changes. According to Table 5, FWRNs produced from KIM and GC rice had higher hardness values (3936.65 and 3812.54 g, respectively), whereas the counterparts prepared with GSLS rice exhibited the lowest hardness value (1237.08 g). This result could be attributed to their high amylose content when compared with other rice varieties (shown in Table 2). Hardness was primarily determined by the rigid helical structures during the cooling process. The continuous water presence in FWRNs further promotes stronger amylose network formation, which aligns with Zhou’s observation that the crystallization structure among amylose and amylopectin during retrogradation would be promote when the water content was proper [28]. The lower amylose content and higher chalkiness (indicative of loosely packed starch granules) of GSLS rice noodles leads to a softer texture, which was supported by its lower hardness values. GC, KIM, and IZG rice noodles had higher elasticity and chewiness, which could be attributed to the synergy effect of protein and chalkiness. High levels of proteins would contribute to a denser and more stable gel network by forming cross-links with starch molecules during gelatinization [8], which would contribute to the chewiness. In addition, KIM and GC had lower gel consistency and higher chalkiness (suggesting structural irregularities), as shown in Table 2, which may contribute to a more elastic but less adhesive texture. This aligns with the report by Cao [29], who found that protein–starch interactions enhance elasticity in rice noodles by forming a more cohesive matrix. These findings highlight the importance of selecting rice varieties with optimal amylose and protein content for desired noodle texture. Future studies could explore the impact of processing parameters (e.g., hydration) on these properties.

#### 2.3.3. Sensory Evaluation of FWRNs Produced from Different Rice Varieties

**Figure 3:**
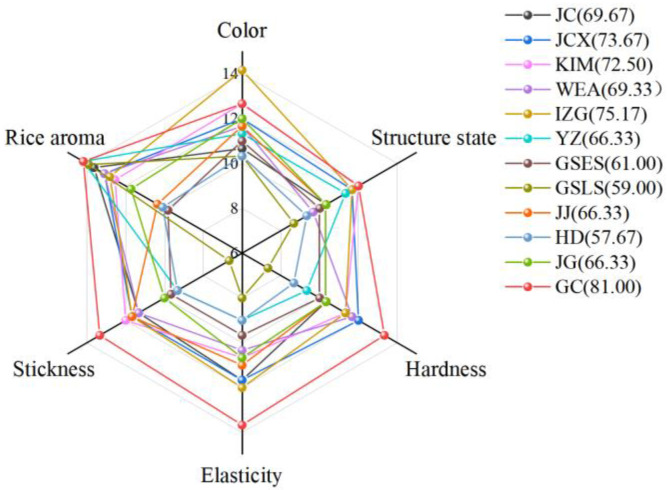
Radar map of the sensory quality of fresh wet rice noodles. Different rice varieties: Jicheng (JC), Jinchangxiang (JCX), Wang’er’ai (WEA), Yuanzao (YZ), Kaima (KIM), India Zhengui (IZG), Guishan early-season (GSES), Guishan late-season (GSLS), Jiajia (JJ), Hengda (HD), Jingu (JG), Guichao (GC). Data in the list is the total sensory score of fresh wet rice noodles from different rice varieties.

According to the sensory scoring standards, sensory quality evaluation effectively determines the appearance, flavor, and edible quality characteristics of food. This method features comprehensive evaluation indicators, clear and detailed descriptions of the scoring criteria, and can verify instrumental quantitative indicators, such as color and texture [30]. The panelists (*n* = 9) were trained professionals in the sensory evaluation of rice noodles, selected based on their ability to discriminate texture, flavor, and appearance attributes. While nine panelists met preliminary screening needs, future work will expand the number to ≥15 panelists for robust statistical power. As illustrated in Figure 3, IZG rice had the highest color score (brighter), followed by GC and GSLS rice. Additionally, the FWRNs produced from KIM and GC rice had a relatively good structural state. The three taste scores (viscosity, elasticity, and hardness) of GC rice noodles were higher than those of other rice noodles, with a moderate hardness and smooth taste. In contrast, the taste score of GSLS rice noodles was the lowest, characterized by softness, rotten odor, and stickiness. FWRNs made from YZ and GC rice had higher aroma scores, although the difference was not significant. After summarizing the appearance, taste, and flavor scores, it was determined that FWRNs produced with GC rice had the highest total sensory score of 81.00, followed by IZG (75.17), JCX (73.67), and KIM (72.50) rice. Meanwhile, FWRNs produced with GSLS and HD rice had the lowest total sensory scores (59.00 and 57.67, respectively).

### 2.4. Correlation Analysis

#### 2.4.1. Correlation of FWRN Quality and Rice Composition

**Figure 4:**
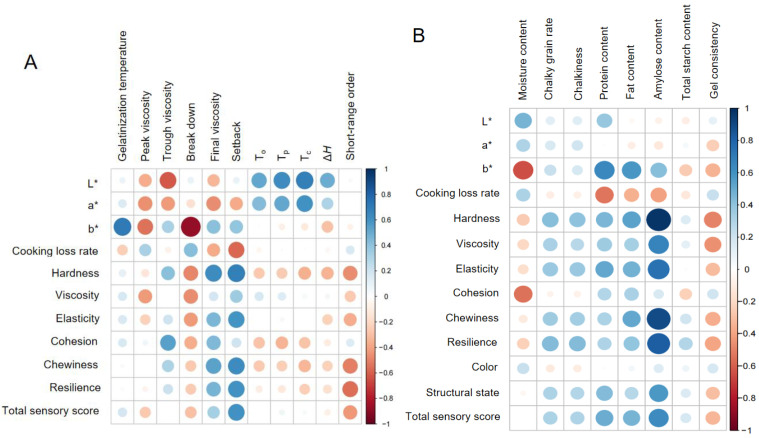
Correlation analysis between rice structural properties and the quality of fresh and wet rice noodles. (**A**): rice compositions and the quality of fresh and wet rice noodles; (**B**): rice starch properties and the quality of fresh and wet rice noodles.

As shown in Figure 4A, amylose content exhibits a highly significant correlation with nearly all structural parameters of FWRNs. It is significantly positively correlated with the hardness, elasticity, chewiness, and resilience of FWRNs and significantly negatively correlated with the cooking loss rate of FWRNs. Moisture content has significant positive and negative correlations with the L* value and b* value, respectively. The chalky grain rate and chalkiness are significantly positively correlated with the amylose content of rice as well as the hardness, elasticity, and resilience of FWRNs. FWRNs made from rice with a higher chalky grain rate tend to be chewier and more elastic. In addition, significant positive correlation is observed between the protein content of rice and the hardness and elasticity of FWRNs. Higher rice protein content benefits the texture and sensory quality of rice noodles, as does fat content. Since gel consistency is significantly negatively correlated with amylose content, it is generally negatively correlated with the quality indicators of FWRNs. Therefore, the amylose content, gel consistency, chalkiness, and protein content are selected as the important indicators affecting the quality of FWRNs, and the combination of amylose content and gel consistency can be considered key indicators for screening rice raw materials for FWRNs.

#### 2.4.2. Correlation of FWRN Quality and the Structural Properties of Rice Starch

As shown in Figure 4B, the peak viscosity of rice starch was significantly negatively correlated with the stickiness (−0.426 **) of FWRNs, whereas trough viscosity was significantly positively correlated with hardness (0.417 **) and cohesion (0.543 **). The breakdown value of rice starch was significantly negatively correlated with the texture characteristics of FWRNs, such as hardness (−0.487 **), viscosity (−0.464 **), and elasticity (−0.425 **). The setback value was significantly positively correlated with the hardness (0.680 **), viscosity (0.353 **), elasticity (0.596 **), chewiness (0.624 **), recovery (0.606 **), and total sensory scores (0.609 **) and was extremely significantly negatively correlated with the cooking loss rate (−0.577 **), indicating that FWRNs with a higher short-term degree of retrogradation would have a better taste and lower cooking loss. Furthermore, the thermal temperature and enthalpy value of rice starch were slightly negatively correlated with the hardness of FWRNs. The surface structure was negatively correlated with the texture characteristics (hardness, viscosity, elasticity, chewiness, recovery) and total sensory scores of FWRNs, indicating that a loose surface structure improved chewiness. Therefore, the setback value and short-range order of rice starch could be selected as indicators of textural properties of FWRNs.

## 3. Conclusions

In this study, the basic composition and physicochemical properties of 12 representative rice varieties produced in southern China were assessed. The sensory, textural, and cooking quality characteristics of FWRNs were subsequently evaluated to establish correlations with the structural properties of rice. The KIM, GC, and IZG rice varieties had a high chalky grain rate and low gel consistency, while the related starches had a high amylose content, high setback value, and low short-range order. The noodles were bright white, achieved high total sensory scores, and exhibited high levels of hardness, springiness, chewiness, and resilience, indicating superior sensory and textural qualities. GSLS rice, which had the lowest amylose and protein contents, exhibited the highest gel consistency, a low setback value, and high short-range order. FWRNs produced with GSLS rice had the poorest sensory quality, characterized by the highest cooking loss; low hardness, springiness, chewiness, and resilience; and high adhesiveness. Correlation analysis revealed that the chalky grain rate, degree of chalkiness, protein and fat contents, and amylose content were significantly and positively correlated with the hardness, elasticity, chewiness, and total sensory score of FWRNs. Conversely, gel consistency showed a significant negative correlation. The setback and surface structure of rice starch positively and negatively affected the texture and sensory quality of the noodles, respectively. Overall, KIM and GC were the better rice varieties for preparation of FWRNs with the best comprehensive quality. Based on the structural parameters of the KIM and GC rice varieties, amylose content, ranging from 26 to 28%, combined with a gel consistency of 33 to 36 mm, would be the key criteria for selecting raw rice for the production of FWRNs with superior quality. These results offer theoretical guidance for rice selection in the industrial-scale production of FWRNs. However, there are still some limitations that need to be addressed in future studies. By expanding sensory evaluations, exploring microstructural mechanisms, and testing diverse culinary applications, future studies could provide more comprehensive guidelines for improving the quality of FWRNs.

## 4. Materials and Methods

### 4.1. Materials

In total, 12 rice varieties were tested. Jicheng (JC) and Jinchangxiang (JCX) rice were purchased from Guangdong Changxiang Grain Co., Ltd. (Dongguan, China). Wang’er’ai, Yuanzao (YZ), Kaima (KIM), and India Zhengui (IZG) rice were imported from Chennai, Tamil Nadu, India. Guishan early-season (GSES), Guishan late-season (GSLS), Jiajia (JJ), Hengda (HD), and Jingu (JG) rice were purchased from Guangdong Bawanghua Rice Noodle Co., Ltd. (Xiangtan, China). Guichao (GC) rice was purchased from Zhaoqing Yongyi Food Co., Ltd. (Zhaoqing, China). A total starch determination kit (K-TSTA) and D-glucose detection kit (K-GLUC) were acquired from Megazyme Ltd. (Bray, Ireland). All chemical reagents were analytical grade.

### 4.2. Measurements of Rice Quality

#### 4.2.1. Chalkiness, Chalky Grain Rate, and Protein, Lipid and Starch Contents

The rice chalkiness rate and chalkiness were measured with a JMWT12 rice appearance quality tester (Dongfujiuheng Instrument Technology Co., Ltd., Beijing, China). The chalky grain rate (%) was calculated as the number of chalky grains/number of observed grains × 100 and chalkiness (%) as the chalky area/total area of observed grains × 100.

Protein content was measured from the total nitrogen content of head rice with a conversion index of 5.95, in accordance with the protocol of Tao et al. [31]. Starch content was quantified using the Megazyme K-TSTA total starch determination kit, which is designed for the measurement of total starch in cereal flours and food products, in accordance with AOAC International method 996.11 and AACC International method 76-13.01 [32].

#### 4.2.2. Gel Consistency

The gel consistency of rice was determined as follows. Rice flour (100 g) and ethanol thymol blue solution (0.2 mL, 95% *w*/*w*) were added to test tubes, which were gently shaken to ensure that the rice flour was fully dispersed without forming agglomerations. After the addition of KOH solution (2.0 mL, 0.2 mol/L), the tubes were heated in a boiling water bath for 8 min, then removed and cooled at room temperature for 6 to 10 min, followed by cooling in an ice water bath for 20 min. The test tubes were then positioned horizontally for 1 h, and the length of the rice gel was recorded.

### 4.3. The Characteristics of Rice Starch

#### 4.3.1. Isolation of Rice Starch

Rice starch was isolated from the rice samples using the method described by Li et al. [33] with minor modifications. Specifically, rice (100 g) was soaked in a sodium metabisulfite solution (0.45%, *w*/*v*) at 4 °C for 16 h and then milled for 5 min with a homogenizer (FK-2; Changzhou Chaoli Homogenizing Pump Factory, Changzhou, China) in an ice water bath. After the addition of NaCl solution (450 mL, 0.1 mol/L) and toluene (50 mL), the mixture was stirred for 2 h and then allowed to stand until the starch precipitated to the bottom and the protein floated to the top. The protein layer was then carefully removed. Finally, the obtained starch precipitate was washed with deionized water and then dried in an oven at 40 °C.

#### 4.3.2. Pasting Properties of Rice Starches

The pasting properties of the rice starches were determined using a Rapid Visco Analyzer (RVA-Starch Master 2; Perten Instruments, Hägersten, Sweden). Briefly, starch (3 g, dry weight) was combined with MilliQ water (25 g) in an RVA canister. The samples were maintained at 50 °C for 1 min, heated to 95 °C for 3.7 min, held at 95 °C for 2.5 min, cooled to 50 °C for 3.8 min, and held at 50 °C for 2 min using a programmed heating and cooling cycle. The parameters of peak viscosity, trough viscosity, final viscosity, breakdown, and setback were measured in centipoise (cP) units.

#### 4.3.3. Thermal Properties of Rice Starches

The thermal properties of the starches were investigated using a differential scanning calorimeter (DSC-3; Mettler-Toledo International Inc., Columbus, OH, USA), as described by Li et al. [34] with slight modifications. Rice starch (3.0 mg, dry weight) was added to distilled water (8 μL) in an aluminum pan, which was hermetically sealed, balanced at room temperature for 12 h, and then heated from 25 to 100 °C at a rate of 10 °C/min. The Universal Analysis program (TA Instruments, New Castle, DE, USA) was used to calculate the temperature at the onset (To), peak (Tp), and conclusion (Tc), in addition to enthalpy change (ΔH).

#### 4.3.4. X-Ray Diffraction (XRD)

The starch crystalline structure was characterized with an X-ray diffractometer (SmartLab SE; Rigaku Corporation, Tokyo, Japan) operating at 40 kV and 40 mA, as described in a previous report [35] with slight modifications. Scans were conducted with the 2θ configuration from 4° to 40°, with a step interval of 0.0525° and a scan rate of 2°/min. Before the scans, all starch samples were equilibrated in a desiccator under a humidified atmosphere of 100% for 48 h. Relative crystallinity (%) was calculated using JADE software (https://www.icdd.com/mdi-jade/, accessed on 26 July 2025; International Centre for Diffraction Data, Newtown Square, PA, USA).

#### 4.3.5. Fourier Transform Infrared Spectroscopy

Material analysis and identification were performed with an FTIR spectrometer (Spectrum 100 series; PerkinElmer, Inc., Shelton, CY, USA; Nicolet IS50; Thermo Fisher Scientific, Waltham, MA USA) equipped with an attenuated total reflectance single reflectance cell featuring a diamond crystal. Each spectrum was obtained from 32 scans conducted at room temperature, spanning the range of 1200 to 800 cm^−1^ with a resolution of 4 cm^−1^. The absorbance ratio at wave numbers 1047 and 1022 cm^−1^ was calculated to represent the ordered short-range structure.

### 4.4. Quality Assessment of FWRNs

#### 4.4.1. Preparation of FWRNs

Dehulled rice was soaked for 2.5 h at room temperature. After the water was removed, fresh water was added to the soaked, dehulled rice to adjust the rice-to-water ratio to 1:2.5. The mixture was then ground and passed through an 80-mesh sieve. After thorough mixing, the slurry (120 g) was spread on a plate in the steamer (JYJ Sanding Rice Noodle Roll Machine, Dongguan, China) and heated to 100 °C for 90 s. After cooling, the FWRNs were cut into strips (length, 20 cm; width, 9 mm) for further use.

#### 4.4.2. Color Analysis of FWRNs

The color characteristics of FWRNs were determined with a colorimeter (CR-400; Konica Minolta, Inc., Osaka, Japan). Measurements of each sample were conducted five times at randomly distributed locations, and the L* (white/black), a* (red/green), and b* (yellow/blue) values were recorded. The brown index was also evaluated as BI = [100 × (x − 0.31)]/0.17, where x = (a* + 1.75 L*)/(5.645 L*+ a* − 3.012 b*).

#### 4.4.3. Cooking Quality of FWRNs

To determine the cooking loss rate of the FWRNs, the moisture content (ω) of each sample (15 g, m_0_/g) was measured. Briefly, rice noodles were boiled in 450 mL of water for 4 min. The volume was maintained at 450 mL. Then, 50 mL was drawn into a vessel (m_1_/g), heated to 105 ± 2 °C, and dried to a constant weight (m_2_/g). The cooking loss rate (%) was calculated as (10 × (m_2_ − m_1_))/(m_0_ × (1 − ω)) × 100 (3).

#### 4.4.4. Texture Determination of FWRNs

The texture of FWRNs was evaluated with a TA–XT2i texture analyzer (Stable Micro System, Ltd., London, UK) in accordance with the modified method of Cham and Suwannaporn [8]. The prepared FWRNs were cut into pieces (length, 5 cm). Subsequently, each set of three layers was measured and positioned at the center of the stage of the texture analyzer. The velocity before, during, and after measurement, the compression ratio, the time interval between two compressions, and the trigger force of the texture analyzer were set to 2 mm/s, 1 mm/s, 1 mm/s, 50%, 3.0 s, and 5 g, respectively. The texture measurement indices (hardness, adhesiveness, elasticity, and chewiness) of each sample were measured five times.

#### 4.4.5. Sensory Evaluation

Sensory evaluation, which plays an indispensable role in the food industry [36], is the measurement, analysis, and interpretation of the interactions between food and other substances, which can be assessed by taste, touch, sight, smell, and hearing because quantitative indicators measured by chemical methods cannot accurately explain the interactions among various sensory elements. The effectiveness of “fuzzy logic” in the sensory evaluation of food products is considered acceptable when the quality aspects perceived by consumers are difficult to correlate with the responsible chemical or physical component attributes [37]. With the fuzzy approach, individuals are requested to provide feedback on the range of intensity on a scale ranging from 0 to 100 [38]. In this study, sensory evaluation was conducted using the method described by Lei et al. [39]. A sensory evaluation team consisting of nine panelists conducted sensory evaluation of rice noodles, as detailed in Table 6. The panelists (*n* = 9) were trained professionals in the sensory evaluation of rice noodles, selected based on their ability to discriminate texture, flavor, and appearance attributes. The evaluation focused on freshly prepared FWRN samples without any kind of soup to standardize the assessment, as this is a common preparation method in southern China. The highest and lowest scores were discarded, and the average was calculated.

### 4.5. Data Processing and Analysis

The data were compared by analysis of variance and the Duncan multiple comparison test with IBM SPSS Statistics for Windows (version 26; IBM Corporation, Armonk, NY, USA). A probability (*p*) value < 0.05 was considered statistically significant.

## Figures and Tables

**Table 1:** The composition and properties of different rice varieties ^1^.

Samples ^2^	Moisture Content/%	Chalky Grain Rates/%	Chalkiness /%	Protein Content/%	Lipid Content /%	Amylose Content/%	Total Starch Content/%	Gel Consistency
JC	11.48 ± 0.04 ^hi^	54.33 ± 1.35 ^cd^	22.97 ± 0.21 ^c^	7.46 ± 0.14 ^cd^	0.26 ± 0.04 ^ef^	22.61 ± 0.61 ^f^	86.07 ± 1.35 ^bcd^	36.57 ± 3.87 ^hi^
JCX	11.51 ± 0.06 ^hi^	31.57 ± 0.57 ^fg^	10.43 ± 0.40 ^g^	7.62 ± 0.23 ^c^	0.84 ± 0.03 ^a^	24.09 ± 0.02 ^e^	84.09 ± 1.94 ^defg^	37.77 ± 4.04 ^ghi^
KIM	12.19 ± 0.27 ^e^	82.53 ± 0.91 ^a^	35.43 ± 0.65 ^a^	6.68 ± 0.16 ^g^	0.31 ± 0.04 ^def^	27.66 ± 0.46 ^a^	90.44 ± 1.70 ^a^	33.97 ± 2.80 ^i^
WEA	11.72 ± 0.11 ^fg^	17.33 ± 1.40 ^h^	5.63 ± 0.51 ^h^	7.17 ± 0.07 ^f^	0.78 ± 0.04 ^ab^	23.81 ± 0.06 ^ef^	84.33 ± 1.19 ^cdefg^	66.83 ± 3.72 ^b^
IZG	12.56 ± 0.10 ^d^	14.73 ± 2.39 ^h^	4.13 ± 0.64 ^h^	7.20 ± 0.03 ^ef^	0.35 ± 0.07 ^cde^	25.58 ± 0.96 ^cd^	83.43 ± 0.37 ^defgh^	60.37 ± 3.01 ^d^
YZ	11.61 ± 0.04 ^gh^	52.43 ± 1.14 ^de^	17.57 ± 0.95 ^de^	8.98 ± 0.13 ^a^	0.45 ± 0.01 ^c^	22.62 ± 0.30 ^g^	80.83 ± 0.28 ^h^	60.60 ± 3.30 ^cd^
GSES	12.91 ± 0.05 ^b^	48.63 ± 12.43 ^de^	19.17 ± 5.36 ^d^	7.14 ± 0.08 ^f^	0.32 ± 0.16 ^def^	24.05 ± 1.43 ^e^	84.52 ± 1.14 ^cdefg^	41.40 ± 3.82 ^fgh^
GSLS	12.74 ± 0.04 ^c^	15.57 ± 0.40 ^h^	5.03 ± 0.06 ^h^	5.48 ± 0.05 ^i^	0.10 ± 0.02 ^gh^	14.61 ± 0.21 ^j^	84.42 ± 1.41 ^cdefg^	77.33 ± 1.20 ^a^
JJ	12.54 ± 0.06 ^d^	43.37 ± 14.72 ^e^	15.03 ± 5.65 ^ef^	7.19 ± 0.13 ^f^	0.30 ± 0.03 ^ef^	21.77 ± 1.41 ^gh^	85.84 ± 0.42 ^bcde^	48.10 ± 3.17 ^e^
HD	12.60 ± 0.03 ^cd^	15.73 ± 0.81 ^h^	4.20 ± 0.26 ^h^	6.27 ± 0.08 ^h^	0.08 ± 0.01 ^h^	16.60 ± 0.33 ^i^	85.03 ± 1.77 ^bcdef^	38.13 ± 3.56 ^ghi^
JG	12.17 ± 0.07 ^e^	61.97 ± 5.36 ^bc^	27.23 ± 3.21 ^b^	7.40 ± 0.07 ^de^	0.25 ± 0.11 ^ef^	21.05 ± 0.77 ^h^	84.77 ± 1.63 ^bcdefg^	65.63 ± 3.41 ^bc^
GC	13.49 ± 0.03 ^a^	81.87 ± 0.97 ^a^	34.53 ± 0.83 ^a^	8.31 ± 0.04 ^b^	0.31 ± 0.02 ^def^	26.81 ± 0.04 ^ab^	87.06 ± 0.40 ^bc^	35.87 ± 2.44 ^i^

^1.^ Different rice varieties: Jicheng (JC), Jinchangxiang (JCX), Wang’er’ai (WEA), Yuanzao (YZ), Kaima(KIM), India Zhengui (IZG), Guishan early-season (GSES), Guishan late-season(GSLS), Jiajia (JJ), Hengda (HD), Jingu (JG), Guichao (GC). ^2.^ Different letters (a–j) in the same column indicate significant differences (*p* < 0.05).

**Table 2:** Gelatinization characteristic values of different raw rice starches ^1^.

Samples ^2^	Pasting Temperature (°C)	Peak Viscosity (cP)	Trough Viscosity (cP)	Final Viscosity (cP)	Breakdown (cP)	Set Back (cP)
JC	86.22 ± 0.55 ^c^	2889.33 ± 35.74 ^g^	2528.67 ± 41.14 ^c^	4614.00 ± 39.13 ^e^	360.67 ± 5.69 ^hi^	2085.33 ± 30.92 ^e^
JCX	88.37 ± 0.03 ^a^	2693.33 ± 27.79 ^h^	2538.00 ± 30.81 ^c^	4645.00 ± 50.57 ^de^	155.33 ± 7.09 ^j^	2107.00 ± 41.39 ^e^
KIM	80.52 ± 0.42 ^i^	3530.00 ± 73.18 ^cd^	2387.33 ± 58.48 ^de^	4737.67 ± 57.59 ^d^	1142.67 ± 16.44 ^bc^	2350.33 ± 7.51 ^d^
WEA	83.28 ± 0.46 ^e^	4104.33 ± 68.89 ^a^	3332.67 ± 93.40 ^a^	5884.67 ± 64.28 ^a^	771.67 ± 91.70 ^f^	2552.00 ± 53.56 ^b^
IZG	79.52 ± 0.08 ^j^	3603.67 ± 51.62 ^bc^	2456.00 ± 44.51 ^cd^	4738.00 ± 36.66 ^d^	1147.67 ± 13.01 ^b^	2282.00 ± 11.53 ^d^
YZ	86.97 ± 0.51 ^bc^	3081.00 ± 14.42 ^f^	2726.67 ± 11.02 ^b^	5181.33 ± 79.25 ^c^	354.33 ± 25.32 ^hi^	2454.67 ± 89.67 ^c^
GSES	83.18 ± 0.51 ^ef^	3175.33 ± 7.57 ^e^	2511.67 ± 24.58 ^c^	4433.33 ± 5.13 ^f^	663.67 ± 19.35 ^fg^	1921.67 ± 22.81 ^f^
GSLS	81.37 ± 0.55 ^h^	4115.00 ± 12.29 ^a^	2192.67 ± 48.26 ^f^	3551.33 ± 36.36 ^i^	1922.33 ± 50.57 ^a^	1358.67 ± 13.87 ^i^
JJ	82.93 ± 0.49 ^efg^	3209.00 ± 11.27 ^e^	2481.67 ± 12.06 ^cd^	4576.67 ± 38.89 ^e^	727.33 ± 4.51 ^f^	2095.00 ± 27.73 ^e^
HD	84.33 ± 0.06 ^d^	3222.33 ± 30.29 ^e^	2303.67 ± 36.14 ^e^	3978.33 ± 46.69 ^h^	918.67 ± 19.69 ^e^	1674.67 ± 11.02 ^h^
JG	82.17 ± 0.42 ^g^	3454.33 ± 24.21 ^d^	2502.00 ± 35.79 ^c^	4285.33 ± 17.04 ^g^	952.33 ± 31.18 ^de^	1783.33 ± 25.54 ^g^
GC	82.45 ± 0.43 ^fg^	3643.67 ± 132.30 ^b^	2700.67 ± 76.58 ^b^	5383.67 ± 84.39 ^b^	943.00 ± 141.59 ^de^	2683.00 ± 75.03 ^a^

^1.^ Different rice varieties: Jicheng (JC), Jinchangxiang (JCX), Wang’er’ai (WEA), Yuanzao (YZ), Kaima (KIM), India Zhengui (IZG), Guishan early-season (GSES), Guishan late-season (GSLS), Jiajia (JJ), Hengda (HD), Jingu (JG), Guichao (GC). ^2.^ Different letters in the same column indicate significant differences (*p* < 0.05).

**Table 3:** Thermal properties and surface order degree of different rice starches ^1^.

Sample ^2^	*T*_o_ (°C)	*T*_p_ (°C)	*T*_c_ (°C)	Δ*H* (J/g)	Short-Range Order (1045 cm^−1^/1022 cm^−1^)
JC	74.30 ± 0.22 ^a^	78.44 ± 0.34 ^d^	84.29 ± 0.36 ^d^	8.74 ± 0.26 ^i^	3.72 ± 0.02 ^d^
JCX	65.16 ± 0.42 ^i^	69.78 ± 0.52 ^j^	74.35 ± 0.32 ^i^	9.98 ± 0.22 ^g^	4.14 ± 0.04 ^a^
KIM	73.41 ± 0.46 ^d^	77.50 ± 0.44 ^f^	82.52 ± 0.24 ^e^	12.52 ± 0.30 ^b^	2.13 ± 0.10 ^h^
WEA	64.51 ± 0.14 ^k^	69.22 ± 0.09 ^j^	74.62 ± 0.14 ^i^	8.55 ± 0.27 ^ij^	3.69 ± 0.02 ^d^
IZG	71.21 ± 0.04 ^f^	76.22 ± 0.09 ^g^	81.39 ± 0.26 ^f^	11.66 ± 0.35 ^bcde^	3.81 ± 0.06 ^cd^
YZ	73.72 ± 0.21 ^cd^	77.95 ± 0.25 ^e^	84.34 ± 0.13 ^d^	12.25 ± 1.01 ^bc^	3.86 ± 0.29 ^bcd^
GSES	73.37 ± 0.05 ^d^	79.11 ± 0.10 ^a^	86.29 ± 0.28 ^b^	11.11 ± 0.32 ^e^	3.97 ± 0.14 ^abc^
GSLS	69.85 ± 0.32 ^g^	74.25 ± 0.42 ^h^	77.15 ± 0.32 ^g^	8.98 ± 0.22 ^i^	4.06 ± 0.14 ^ab^
JJ	74.19 ± 0.12 ^ab^	79.00 ± 0.17 ^abc^	85.31 ± 0.14 ^c^	11.63 ± 0.28 ^bcde^	3.73 ± 0.10 ^cd^
HD	67.25 ± 0.32 ^h^	73.15 ± 0.42 ^i^	76.85 ± 0.52 ^h^	9.25 ± 0.22 ^h^	3.68 ± 0.16 ^d^
JG	73.71 ± 0.16 ^d^	78.72 ± 0.19 ^bcd^	87.57 ± 0.52 ^a^	14.65 ± 0.24 ^a^	2.51 ± 0.10 ^f^
GC	72.31 ± 0.32 ^e^	78.67 ± 0.29 ^cd^	85.22 ± 0.40 ^c^	10.15 ± 0.56 ^f^	2.14 ± 1.17 ^h^

^1.^ Different rice varieties: Jicheng (JC), Jinchangxiang (JCX), Wang’er’ai (WEA), Yuanzao (YZ), Kaima(KIM), India Zhengui (IZG), Guishan early-season (GSES), Guishan late-season (GSLS), Jiajia (JJ), Hengda (HD), Jingu (JG), Guichao (GC). ^2.^ Different letters in the same column indicate significant differences (*p* < 0.05).

**Table 4:** The color and cooking quality of fresh wet rice noodles prepared from different rice varieties ^1^.

Samples ^2^	L *	a *	b *	Brown Index	Cooking Loss Rate/%
JC	69.55 ± 0.32 ^de^	2.57 ± 0.02 ^cd^	−0.80 ± 0.08 ^b^	1.52 ± 0.10 ^a^	0.12 ± 0.01 ^efg^
JCX	70.93 ± 0.87 ^cd^	2.56 ± 0.01 ^cd^	−1.48 ± 0.04 ^cd^	0.56 ± 0.05 ^b^	0.12 ± 0.03 ^efg^
KIM	69.28 ± 1.14 ^e^	2.24 ± 0.07 ^hi^	−2.38 ± 0.02 ^g^	−0.98 ± 0.13 ^e^	0.11 ± 0.01 ^efg^
WAA	66.52 ± 1.25 ^fg^	2.17 ± 0.10 ^i^	−1.27 ± 0.03 ^c^	0.48 ± 0.10 ^b^	0.10 ± 0.01 ^fg^
IZG	73.97 ± 1.17 ^ab^	2.30 ± 0.04 ^ghi^	−2.36 ± 0.20 ^g^	−0.84 ± 0.08 ^e^	0.13 ± 0.01 ^defg^
YZ	75.04 ± 0.16 ^a^	2.16 ± 0.05 ^i^	−0.49 ± 0.25 ^ab^	1.42 ± 0.15 ^a^	0.07 ± 0.01 ^g^
GSES	73.39 ± 0.96 ^b^	3.17 ± 0.03 ^a^	−1.90 ± 0.22 ^ef^	0.57 ± 0.13 ^b^	0.34 ± 0.20 ^b^
GSLS	71.75 ± 0.21 ^c^	2.35 ± 0.07 ^fgh^	−3.54 ± 0.23 ^h^	−2.36 ± 0.10 ^f^	0.49 ± 0.01 ^a^
JJ	71.65 ± 0.20 ^c^	2.64 ± 0.01 ^c^	−1.75 ± 0.08 ^de^	0.27 ± 0.05 ^c^	0.19 ± 0.01 ^cdefg^
HD	67.69 ± 0.43 ^f^	2.53 ± 0.20 ^cde^	−2.12 ± 0.42 ^fg^	−0.34 ± 0.03 ^d^	0.14 ± 0.01 ^defg^
JG	71.59 ± 0.61 ^c^	2.80 ± 0.02 ^b^	−2.32 ± 0.13 ^g^	−0.32 ± 0.08 ^d^	0.20 ± 0.01 ^cdefg^
GC	74.59 ± 0.11 ^ab^	2.46 ± 0.03 ^def^	−2.17 ± 0.21 ^fg^	−0.44 ± 0.09 ^d^	0.18 ± 0.01 ^cdefg^

^1.^ Different rice varieties: Jicheng (JC), Jinchangxiang (JCX), Wang’er’ai (WEA), Yuanzao (YZ), Kaima (KIM), India Zhengui (IZG), Guishan early-season (GSES), Guishan late-season (GSLS), Jiajia (JJ), Hengda (HD), Jingu (JG), Guichao (GC). ^2.^ Different letters in the same column indicate significant differences (*p* < 0.05).

**Table 5:** Texture characteristics of fresh and wet rice noodles ^1^.

Samples ^2^	Hardness (g)	Stickiness (g·s)	Elasticity	Cohesion	Chewiness	Resilience
JC	2827.26 ± 72.40 ^e^	−629.30 ± 226.22 ^bcd^	0.77 ± 0.01 ^a^	0.64 ± 0.06 ^bcdef^	2477.61 ± 118.83 ^hi^	0.16 ± 0.01 ^e^
JCX	3265.86 ± 9.00 ^cd^	−635.03 ± 162.09 ^bcd^	0.74 ± 0.01 ^bcd^	0.73 ± 0.02 ^ab^	3527.59 ± 49.24 ^de^	0.17 ± 0.01 ^de^
KIM	3936.65 ± 102.35 ^a^	−260.51 ± 15.46 ^a^	0.70 ± 0.01 ^cdef^	0.56 ± 0.07 ^ef^	4372.86 ± 418.35 ^a^	0.26 ± 0.02 ^a^
WAA	3149.90 ± 57.82 ^d^	−985.69 ± 123.37 ^f^	0.72 ± 0.03 ^bcde^	0.73 ± 0.01 ^ab^	3448.54 ± 202.00 ^de^	0.17 ± 0.01 ^de^
IZG	3441.20 ± 134.44 ^b^	−226.50 ± 55.58 ^a^	0.78 ± 0.02 ^ab^	0.58 ± 0.02 ^def^	4060.36 ± 191.27 ^ab^	0.19 ± 0.01 ^cd^
YZ	2822.92 ± 69.54 ^e^	−604.63 ± 271.70 ^bcd^	0.66 ± 0.05 ^ef^	0.72 ± 0.06 ^ab^	2355.51 ± 129.30 ^hi^	0.17 ± 0.01 ^de^
GSES	3280.77 ± 40.58 ^cd^	−601.95 ± 152.73 ^bcd^	0.69 ± 0.05 ^cdef^	0.62 ± 0.01 ^cdef^	2897.94 ± 50.58 ^fg^	0.16 ± 0.02 ^e^
GSLS	1237.08 ± 69.64 ^h^	−1315.54 ± 56.51 ^g^	0.57 ± 0.02 ^g^	0.55 ± 0.01 ^f^	1785.83 ± 217.83 ^j^	0.11 ± 0.02 ^f^
JJ	2274.57 ± 49.25 ^f^	−365.19 ± 125.47 ^ab^	0.65 ± 0.01 ^f^	0.66 ± 0.02 ^abcde^	2456.11 ± 268.10 ^hi^	0.13 ± 0.01 ^f^
HD	1492.42 ± 32.83 ^g^	−1069.32 ± 193.30 ^fg^	0.55 ± 0.03 ^g^	0.55 ± 0.01 ^f^	1106.90 ± 217.35 ^k^	0.10 ± 0.01 ^f^
JG	2181.22 ± 166.16 ^f^	−951.07 ± 45.49 ^ef^	0.68 ± 0.05 ^def^	0.73 ± 0.07 ^ab^	2231.17 ± 162.73 ^i^	0.11 ± 0.01 ^f^
GC	3812.54 ± 109.39 ^a^	−794.75 ± 270.44 ^cdef^	0.81 ± 0.03 ^a^	0.56 ± 0.11 ^ef^	4341.10 ± 111.07 ^a^	0.21 ± 0.03 ^bc^

^1.^ Different rice varieties: Jicheng (JC), Jinchangxiang (JCX), Wang’er’ai (WEA), Yuanzao (YZ), Kaima (KIM), India Zhengui (IZG), Guishan early-season (GSES), Guishan late-season (GSLS), Jiajia (JJ), Hengda (HD), Jingu (JG), Guichao (GC). ^2.^ Different letters in the same column indicate significant differences (*p* < 0.05).

**Table 6:** Criteria for the sensory evaluation of FWRNs.

Level 1 Indicators (Scores)	Secondary Indicators (Scores)	Specific Characteristic Description (Scores)
Appearance (30 points)	Color (15 points)	Uniform color, normal off-white, glossy surface (11–15 points)
		Basically, uniform color, slightly yellowish skin color, glossy surface (6–10 points)
		Uneven color, yellowish skin color, dull surface (1–5 points)
	Structure (15 points)	Uniform and delicate cross-sectional structure, uniform thickness, smooth surface (11–15 points)
		Uniform cross-sectional structure, basically uniform thickness, smooth surface (6–10 points)
		Granules in cross-sectional structure, uneven thickness, and rough surface (1–5 points)
Taste (50 points)	Hardness (15 points)	Moderately soft and hard (11–15 points)
		Softer or harder (6–10 points)
		Too soft or too hard (1–5 points)
	Elasticity (15 points)	Chewy and elastic (11–15 points)
		Slightly chewy and generally elastic (6–10 points)
		Poor chewiness and insufficient elasticity (1–5 points)
	Viscosity (15 points)	Refreshing and not sticky (11–15 points)
		Refreshing and slightly sticky (6–10 points)
		Not refreshing and sticky (1–5 points)
Flavor (20 points)	Rice aroma (20 points)	The rice aroma is strong and pure with no unpleasant odor (16–20 points)
		The rice aroma is relatively light and pure with no unpleasant odor (11–15 points)
		The rice aroma is average with a slight odor of raw noodles (6–10 points)
		No rice aroma with sour or raw noodles odor (1–5 points)

## Data Availability

The data presented in this study are available on request from the corresponding author due to containing information that could compromise the commercial interests and proprietary technology of the research partners.
